# Normative values of motor performance and their relationship with BMI status in Hong Kong preschoolers

**DOI:** 10.1038/s41598-024-57121-y

**Published:** 2024-03-19

**Authors:** Ka-Man Yip, Hung-Kwan So, Keith T. S. Tung, Rosa S. Wong, Winnie W. Y. Tso, Ian C. K. Wong, Jason C. Yam, Mike Y. W. Kwan, Lobo H. T. Louie, Albert Lee, Wilfred H. S. Wong, Lai-Ling Hui, E. A. S. Nelson, Patrick Ip

**Affiliations:** 1 Department of Paediatrics and Adolescent Medicine, The University of Hong Kong, Hong Kong, People’s Republic of China; 2 Department of Special Education and Counselling, The Education University of Hong Kong, Hong Kong, People’s Republic of China; 3 Centre for Safe Medication Practice and Research, Department of Pharmacology and Pharmacy, The University of Hong Kong, Hong Kong, People’s Republic of China; 4 Department of Ophthalmology and Visual Sciences, The Chinese University of Hong Kong, Hong Kong, People’s Republic of China; 5 Department of Paediatrics and Adolescent Medicine, Princess Margaret Hospital, Hong Kong, People’s Republic of China; 6 Department of Health and Physical Education, The Education University of Hong Kong, Hong Kong, People’s Republic of China; 7 School of Public Health and Primary Care, The Chinese University of Hong Kong, Hong Kong, People’s Republic of China; 8 Department of Paediatrics and Adolescent Medicine, The Chinese University of Hong Kong, Hong Kong, People’s Republic of China; 9 Department of Food Science and Nutrition, The Hong Kong Polytechnic University, Hong Kong, People’s Republic of China; 10 School of Medicine, The Chinese University of Hong Kong, Shenzhen, 518172 Guangdong People’s Republic of China; 11 Department of Paediatrics and Adolescent Medicine, Hong Kong Children’s Hospital, Hong Kong, People’s Republic of China

**Keywords:** Motor performance, BMI status, Preschoolers, Physiology, Health care, Medical research

## Abstract

This study aimed to establish sex- and age-specific reference values for motor performance (MP) in Hong Kong preschoolers aged 3–5 years old and examine the relationship between MP and BMI status. A cross-sectional study was conducted among 5579 preschoolers in Hong Kong. Three MP tests were administered, and height and weight information were collected. GAMLSS was used to compute the normative values of the motor tests. Boys outperformed girls in activities requiring muscle strength and power, while girls outperformed boys in activities requiring balance and coordination. The MP scores increased with age for both overarm beanbag throw and standing long jump for both sexes, while the one-leg balance scores showed larger differences between P_50_ and P_95_ in older preschoolers. Children with excessive weight performed worse in standing long jump and one-leg balance compared to their healthy weight peers. This study provides valuable information on the MP of preschoolers in Hong Kong, including sex- and age-specific reference values and the association between BMI status and MP scores. These findings can serve as a reference for future studies and clinical practice and highlight the importance of promoting motor skill development in preschoolers, particularly those who are overweight or obese.

## Introduction

Motor performance (MP) is the ability of a child to perform basic gross motor skills, which are movements used in daily life and physical activity^1^. Encouraging motor skills in early childhood may be a practical approach to maintaining engagement in physical activities throughout the stages of childhood and adolescence^2,3^. The preschool years are crucial for improving MP due to the "proficiency barrier," which prevents individuals with low motor skills from engaging in lifelong physical activity^1,3,4^. While physical fitness is regarded as a powerful health indicator in children^5,6^, MP is a component of physical fitness in preschool years that is linked to sports performance and motor skills^7–10^. During this time, children develop fundamental motor skills that they later apply to both organized and unorganized physical activity^11^. These skills are crucial for children to participate in sports as adults^12,13^.

Standardized measurements have been reported for schoolchildren above 6 years old from various populations^14–18^. However, literature addressing reference data of MP among preschoolers (3–5 years old) is rather scarce^7,19–21^. A set of tests for MP in Hong Kong preschoolers was developed 20 years ago^9^. The development of children’s motor skills and coordination in preschoolers has shown signs of a secular decline^22,23^. Recent research linking poor MP in typically-developing preschool children to low levels of physical activity highlights the importance of monitoring typically-developing children using standardized measurements^24^.

Childhood obesity is a significant public health concern as it increases the risk of chronic diseases such as hypertension, type 2 diabetes, stroke, cardiovascular disease, hyperlipidaemia, certain types of cancer, and decreased life expectancy^25^. The World Health Organization reported that approximately 39 million children under the age of 5 were overweight or obese in 2020, and children below 5 years old have shown a rapid increase in the development of obesity in recent years^26^. Previous studies have shown a negative association between school-aged childhood obesity and MP^27–29^. However, the relationship between obesity and MP in preschoolers is unclear^30,31^, and the available data is limited^31–34^. Most previous reports used body mass index (BMI) or percentage of body fat as continuous variables instead of categorical variables to determine the association with MP and coordination in young children, and their sample sizes were small^31,34–37^. Given the importance of obesity and fitness as predictors of health-related factors, it is essential to identify their relationship in preschool years. Additionally, improvements in fitness have been shown to reduce the risk of becoming overweight/obese during adolescence^28,38,39^ and BMI-related impairments in MP start to emerge in preschool age^37,40–42^.

Therefore, this study aimed to provide sex-and age-specific MP reference for preschoolers aged 3–5 years old in Hong Kong. This study also addressed sex-related differences across this age period, as well as the relationship between MP and BMI status in preschoolers.

## Results

### Descriptive characteristics

A total of 5579 preschoolers aged 3–5 years were enrolled in 63 kindergartens, comprising 2916 boys and 2663 girls. The mean age of the study population was 4.5 years. Descriptive characteristics of the participants are presented in Table 1. Boys were found to be taller than girls (105.2 cm vs 103.8 cm, p < 0.001), but there was no significant difference in weight by sex. BMI categories were utilized to classify BMI status, with the majority of children (88.0%) classified as having a healthy weight, 7.7% classified as having an excessive weight (overweight or obese), and 4.3% classified as underweight. BMI status classifications were similar between boys and girls (p = 0.072). The average results of the fitness tests for overarm beanbag throw, standing long jump, and one-leg balance were 299.1 cm, 74.1 cm, and 18.4 s, respectively. Significant sex differences were observed in all MP tests (all p values < 0.001), with boys performing better in overarm beanbag throw (313.6 cm vs 283.3 cm, p < 0.001) and standing long jump (75.7 cm vs 72.4 cm, p < 0.001), and girls performing better in one-leg balance (20.7 s vs 16.2 s, p < 0.001).

**Table 1:** Subjects' descriptive characteristics.

	All (n = 5579)		Boys (n = 2916)		Girls (n = 2663)		p value
	N/mean	%/SD	N/mean	%/SD	N/mean	%/SD	
Age (year)							
3	1630	29.2%	833	28.6%	797	29.9%	0.377
4	2534	45.4%	1324	45.4%	1210	45.4%	
5	1415	25.4%	759	26.0%	656	24.6%	
Height (cm)	104.5	6.6	105.2	6.5	103.8	6.5	< 0.001***
Weight (kg)	16.9	3.0	17.2	3.1	16.6	2.8	< 0.001***
BMI (kg/m^2^)	15.4	1.5	15.46	1.49	15.29	1.47	< 0.001***
BMI status^#^							
Underweight	241	4.3%	127	4.4%	114	4.3%	0.072
Healthy weight	4910	88.0%	2569	88.1%	2341	87.9%	
Excessive weight	428	7.7%	220	7.5%	208	7.8%	
MP tests							
Overarm beanbag throw (cm)	299.1	105.3	313.6	112.3	283.3	94.5	< 0.001***
Standing long jump (cm)	74.1	22.1	75.7	23.0	72.4	20.9	< 0.001***
One-leg balance (sec)	18.4	20.4	16.2	18.6	20.7	22.1	< 0.001***

*MP* motor performance.

*p value < 0.05; ** p value < 0.01; ***p value < 0.001; ^#^BMI status was accessed by IOTF reference.

### The fitted models

Table 2 presents a summary of the fitted models. The effective degrees of freedom (μ) for all models were found to be greater than 2, suggesting that there was no linear relationship between age and medians of fitness test scores for either boys or girls. Additionally, the majority of effective degrees of freedom (ν) were greater than 2, indicating that there was no linear relationship between age and skewness.

**Table 2:** GAMLSS models for MP tests among 3 to 5-year-old Hong Kong children.

Mp test	Distribution	link	df(μ)	*df(σ)*	df(ν)	df(τ)	df	Deviance	AIC	SBC
Boys										
Overarm beanbag throw (cm)	BCT	log	2.00	2.00	2.77	2.00	8.77	34,632.80	34,649.55	34,701.99
Standing long jump (cm)	BCT	log	2.97	2.98	2.00	2.69	10.64	25,306.29	25,325.22	25,388.81
One-leg balance (s)	BCT	log	2.00	2.00	2.97	2.99	9.95	20,640.82	20,660.32	20,719.82
Girls										
Overarm beanbag throw (cm)	BCT	log	2.89	2.85	2.00	2.00	9.74	30,661.81	30,679.55	30,736.91
Standing long jump (cm)	BCT	log	2.98	2.98	2.00	2.00	9.97	22,473.11	22,490.85	22,549.52
One-leg balance (s)	BCPE	log	2.91	3.00	2.00	2.94	10.85	20,170.79	20,191.61	20,255.50

*MP* motor performance.

### Normative values and centile curves of the MP scores

**Figure 1:**
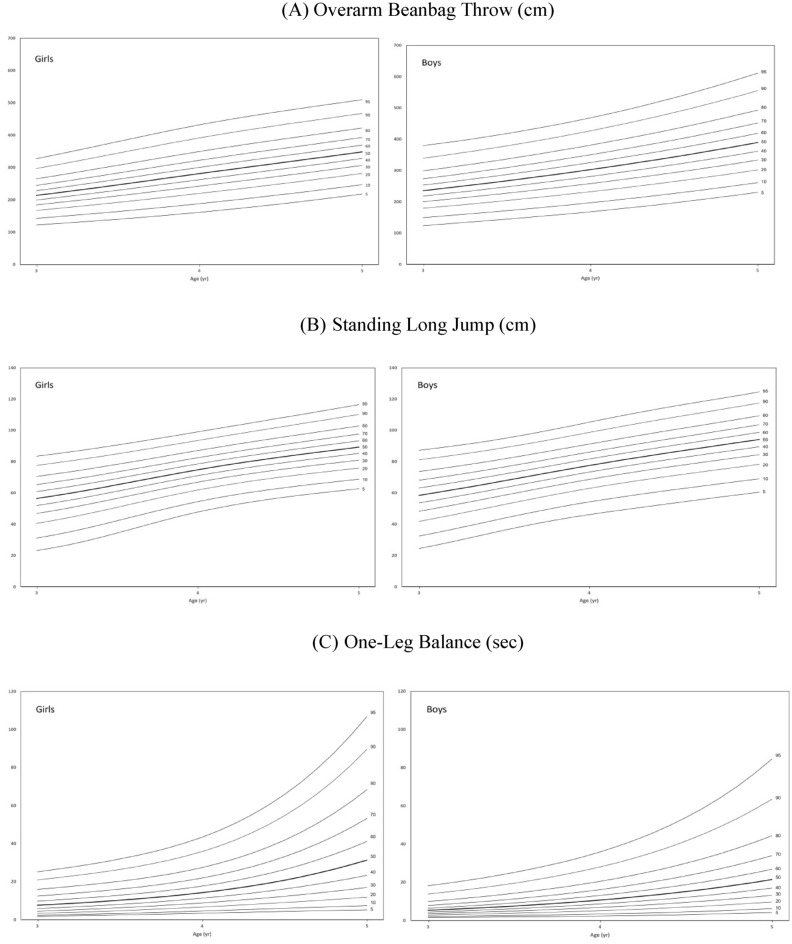
Percentile curves for overarm beanbag throw, standing long jump and one-leg balance for boys and girls.

Table 3a–c provide the normative values of the MP tests presented as percentiles ranging from 5 to 95th Smoothed centile curves (P_5_, P_10_, P_20_, P_30_, P_40_, P_50_, P_60_, P_70_, P_80_, P_90_, and P_95_) relative to an age interval of 1 month for the MP scores by age and sex for children are presented in Fig. 1a–c. The tables and figures demonstrate that boys outperformed girls in the overarm beanbag throw and standing long jump, while girls outperformed boys in the one-leg balance. The MP scores for overarm beanbag throw and standing long jump increased steadily with age for both sexes. In the one-leg balance test, we observed larger differences between P_50_ and P_95_ in older preschoolers than in their younger counterparts (Fig. 1c). The skewness of one-leg balance time increased with age for both boys and girls. Our findings suggest that there are linear changes based on relative age in the various dimensions of tests studied in preschoolers.

**Table 3:** (a) Overarm beanbag throw (cm), (b) standing long jump (cm) and (c) one-leg balance (s) centile values by sex and age in 3 to 5-year-old Hong Kong children.

Age (year)	Model	μ	*σ*	ν	τ	P_5_	P_10_	P_20_	P_30_	P_40_	P_50_	P_60_	P_70_	P_80_	P_90_	P_95_
(a) Overarm beanbag throw (cm)																
Boys																
3	BCT	0.28	235.65	0.55	6.03	123.7	149.5	179.3	200.4	218.5	235.7	253.5	273.5	298.9	339.4	379.6
4	BCT	0.28	302.92	0.59	15.48	168.2	196.9	232.3	258.3	281.1	302.9	325.4	350.4	381.1	426.8	468.3
5	BCT	0.29	389.40	0.31	39.72	230.2	261.3	301.9	333.2	361.5	389.4	418.7	451.7	492.9	555.0	611.2
Girls																
3	BCT	0.26	213.84	0.55	7.70	122.5	143.2	167.4	184.7	199.6	213.8	228.5	244.9	265.4	297.2	327.7
4	BCT	0.26	281.14	0.52	8.24	161.6	188.3	219.9	242.7	262.3	281.1	300.6	322.3	349.5	391.7	432.0
5	BCT	0.23	348.57	0.50	8.82	218.1	247.3	281.9	306.7	328.1	348.6	369.6	393.1	422.3	467.3	509.9
(b) Standing long jump (cm)																
Boys																
3	BCT	0.33	58.11	1.40	174.30	24.5	32.4	41.8	48.3	53.7	58.5	63.2	68.1	73.7	81.2	87.3
4	BCT	0.20	77.47	1.36	9.17	46.2	54.3	62.9	68.7	73.3	77.5	81.7	86.1	91.3	98.7	105.2
5	BCT	0.20	94.17	1.31	10.82	60.5	69.0	78.3	84.5	89.6	94.2	98.7	103.6	109.3	117.5	124.6
Girls																
3	BCT	0.33	55.28	1.72	12.75	23.2	31.2	40.6	46.9	52.0	56.5	60.9	65.4	70.6	77.6	83.5
4	BCT	0.19	74.82	1.32	16.81	47.8	54.5	61.9	66.9	71.1	74.8	78.6	82.5	87.1	93.6	99.0
5	BCT	0.18	89.31	0.92	22.16	62.7	68.8	75.9	81.0	85.3	89.3	93.3	97.7	102.8	110.2	116.5
(c) One-leg balance (s)																
Boys																
3	BCT	0.7	5.36	-0.01	4.70E + 05	1.8	2.4	3.4	4.5	5.9	7.6	9.8	12.5	16.0	21.0	25.2
4	BCT	0.8	10.72	0.17	9.00E + 06	3.4	4.6	6.7	8.9	11.3	14.2	17.7	22.0	27.5	35.9	43.4
5	BCT	0.9	21.44	0.11	1.73E + 08	5.2	7.4	11.8	17.0	23.4	31.4	41.2	53.3	68.4	89.6	106.9
Girls																
3	BCPE	0.8	7.63	0.14	4.15	1.8	2.4	3.4	4.5	5.9	7.6	9.8	12.5	16.0	21.0	25.2
4	BCPE	0.8	14.23	0.20	2.94	3.4	4.6	6.7	8.9	11.3	14.2	17.7	22.0	27.5	35.9	43.4
5	BCPE	0.9	31.36	0.26	4.17	5.2	7.4	11.8	17.0	23.4	31.4	41.2	53.3	68.4	89.6	106.9

### Association between BMI status and MP z-scores

Table 4 presents a comparison of the MP z-scores among BMI status groups. We observed significant differences in the z-scores of all tests among the groups. The mean test scores of children with excessive weight were found to be significantly lower than the other groups (p < 0.001) when compared to children with healthy weight, except for overarm beanbag throw. Children with excessive weight performed best in overarm beanbag throw (p < 0.001) but performed worse in standing long jump (p < 0.001). The one-leg balance score in children with healthy weight was better than those with excessive weight (− 0.196 vs 0.019, p < 0.001).

**Table 4:** Comparison of motor performance z-scores between BMI status groups#.

	Total (n = 5579)		Underweight (n = 241)		Healthy weight (n = 4910)		Excessive weight (n = 428)		F	p value
	Mean	SD	Mean	SD	Mean	SD	Mean	SD		
Fitness z-scores										
Overhead Beanbag Throw	0.005	1.001	− 0.203^a^	0.985	− 0.002^b^	0.998	0.209^c^	1.007	14.321	< 0.001
Standing Long Jump	0.004	0.998	− 0.001^a^	1.016	0.025^a^	0.995	− 0.232^b^	1.000	13.142	< 0.001
One-leg balance	− 0.002	0.999	− 0.099^ab^	0.992	0.019^a^	1.000	− 0.196^b^	0.967	10.383	< 0.001

a, b, and c indicate significant differences between lettered groups by post hoc pairwise comparison.

*p value < 0.05; **p value < 0.01; ***p value < 0.001; ^#^BMI status was accessed by IOTF reference.

## Discussion

This study established sex- and age-specific reference values for MP in Hong Kong preschoolers aged 3–5 years old and examined the relationship between MP and their BMI status. The findings of this study provide valuable information on the MP of preschoolers in Hong Kong, which can serve as a reference for future studies and clinical practice. The results showed that boys have better performance in activities requiring muscle strength and power, while girls have better performance in activities requiring balance and coordination. These findings are consistent with previous studies that have reported sex differences in MP^7–9,43^.

The MP scores increased steadily with age for both overarm beanbag throw and standing long jump for both sexes, which is consistent with previous studies^7,9,30,44^. In older preschool children, the difference in one-leg balance scores between the P_50_ and P_95_ was greater compared to younger preschool children. This finding is similar to the results of a 2019 study conducted on Spanish preschoolers^7^, indicating that children who have not developed good balance skills during their early years may struggle to catch up with their peers as they grow older. Our findings support that both age and sex significantly affect balance skill in preschoolers, as suggested by Franjoine et al*.* in 2010^45^ and Latorre Roman et al*.* in 2017^46^. This highlights the importance of early intervention to promote motor skill development in preschoolers.

The study revealed a significant correlation between BMI status and MP scores, indicating that children with excessive weight performed worse in standing long jump and one-leg balance compared to their healthy weight peers. These findings are consistent with previous studies that have reported a negative association between childhood obesity and MP in school children^28,29,35,47^ and suggest that MP is probably linked to the obesity and weakened musculoskeletal abilities of excessive weight children^32^. Additionally, this finding emphasizes the potential impact of muscular development in reducing the disparity in MP based on BMI status. The observed inconsistencies in our results, such as the lower mean test scores of children with excessive weight compared to those with healthy weight, except for overarm beanbag throw, can be explained by this finding. The ability to jump is a crucial skill that is often utilized in activities that require high energy expenditure. If obese children have limitations in this area, they may be less likely to participate in sports and physical activities that require jumping^48,49^. This reduced engagement in sports and physical activities that involve jumping may contribute to the continued presence of excessive body weight and additional fat accumulation^50^. Overall, the results of this study suggest that the negative association between obesity and MP may already be present in the preschool years.

To interpret the results of the MP assessment, it is necessary to compare the individual score with reference values for the general population, taking into account factors such as sex and age. Therefore, this research provides normative values of the general population, which allows for the assessment to compare the ability of Hong Kong children at defined ages. Physical fitness below P_5_ may be a sign of a potentially pathological condition, according to previous publications^14,17^. The lowest percentile can be used as a ''warning signal'' to perform additional tests to identify potential motor delays. Poor MP may motivate those who are directly responsible, such as parents, doctors, or the school community, to take action.

One limitation of this study is that it was cross-sectional, which limits the ability to establish causality between BMI status and MP scores. Future longitudinal studies are needed to examine the relationship between changes in BMI status and changes in MP scores over time. While BMI is a widely used measure for assessing health risk in preschoolers, it may not provide a complete picture of body composition. Markers of body fat, such as skinfold thickness or bioelectrical impedance, may offer a more accurate assessment of body composition. Nevertheless, the results of this study revealed a correlation between BMI status and MP scores, with overweight children exhibiting poorer performance in standing long jump and one-leg balance compared to healthy weight preschoolers. To determine the sequential relationship between BMI status and MP development, longitudinal studies are necessary. Another limitation is that the study sample was limited to kindergartens in Hong Kong, which may not be representative of the entire population of preschoolers in other regions. Although there is a lack of extensive research on the long-term effects of tested motor performance, the present study provides recommendations on the necessary tests to be conducted and how to obtain valuable reference values. However, further research is needed to fully understand the long-term implications.

## Conclusion

In conclusion, this study provides valuable information on the MP of preschoolers in Hong Kong, including sex- and age-specific reference values and the association between BMI status and MP scores. These findings can serve as a reference for future studies and clinical practice and highlight the importance of promoting motor skill development in preschoolers, particularly those who are overweight or obese. MP is crucial for younger children due to global decline trends in physical activity^22,23^. It is important to prioritize MP at an early age to prevent future health issues and foster a lifelong habit of physical activity.

## Methods

### Participants and data collection

This cross-sectional study was conducted in kindergartens participating in the Hong Kong Growth Study and a contemporary community study, both of which aimed to collect updated data on growth parameters and support children's holistic development. To compile a sampling frame of all kindergartens in Hong Kong, a list of kindergartens from the "Profile of Kindergarten and Kindergarten-cum-child care centres, Education Bureau" was used for the Hong Kong Growth Study. Five kindergartens were randomly selected from each of the 5 major regions in Hong Kong-Hong Kong Island, New Territory East, New Territory West, Kowloon East, and Kowloon West-based on computer-generated random numbers according to the numbers of schools per region. If a selected school declines to participate in a study, the next school in the random selection process is invited to take its place. This ensures that the sample remains representative and helps maintain the integrity of the study.

In this study, all children who belong to the selected classrooms were invited to participate. Three MP tests including the one-leg balance (OB), standing long jump (SLJ) and beanbag throwing (BT) were administered to preschoolers to measure the students’ gross motor development and performance in static balance and muscle strength (both upper and lower limbs). These tests were adapted from the PREFIT fitness battery^51^ and Hong Kong Pre-school Motor Performance Award Scheme^9^. These tests were chosen because they were found to be reliable (r > 0.71) in preschoolers and could represent overall health among young children^9,51^. The study also incorporates data on height and weight. Data collection took place between December 2019 and August 2022. An informed consent form was voluntarily signed by parents allowing their children to take part in the study. The study was conducted in accordance with the Declaration of Helsinki, and approved by Ethics approval for HKGS was obtained from the Joint Chinese University of Hong Kong-New Territories East Cluster Clinical Research Ethics Committee (CREC ref. no. 2019.575), the University of Hong Kong-Hospital Authority Hong Kong West Cluster Joint Institutional Review Board (UW 18-593, UW 17-491) and the Ethics Committee of the Department of Health, Hong Kong SAR Government (LM 307/2018).

### MP tests

Fundamental motor skills such as walking, running, skipping, and rolling play a crucial role in physical fitness, as they contribute to the development of strength, power, and endurance^52^. Seefeldt emphasized the importance of these basic motor skills in performing activities that promote structural and cardiovascular fitness in later years. Therefore, physical fitness tests for this age group should be closely related to and overlap with developmental assessment tests for gross motor skills. Based on a pilot study, the selected tests for this study met the following criteria:

1. Appropriate for children aged 3–6 years.
2. Activities that are simple to learn and safe to perform.
3. Easily administered by a kindergarten teacher.
4. Clearly defined and simple to score.
5. Limited to the use of materials already available in Hong Kong kindergartens.
6. Similar to motor performance tests for kindergarten children aged 3–6 years used in other parts of the world.
7. Covering all aspects of motor performance, including static balance and muscle strength (both upper and lower limbs).

The preschoolers were given standard demonstrations and verbal cues to help them perform better during the actual tests. All tests were carried out in kindergartens and were supervised by a research team, with teachers also present, during playtime with each group of preschoolers. The participants completed all the tests in small groups, typically consisting of around 5 children for each task. Before the tests began, all participants completed a 5-min general and standardized warm-up. Investigators had previously been trained in administering all tests and following standard operating procedures, which included a proficient demonstration of each test technique, a verbal explanation, and motivational feedback.

### One-leg balance

The participants began the test by standing on their dominant foot and placing their arms by their sides, not moving their feet or grabbing a support unless they needed to regain their balance. There were no restrictions on upper-limb movement as per the original protocol. When the participants moved from their stationary position or put the other leg down, the test was stopped. Recorded the best time covered for three trials in seconds for the foot used. The test was timed using a stopwatch. The timing with the stopwatch began when the heel was lifted off the floor. Each child was given 2 practice trials and 3 tests. After 3 tests, the best result was selected.

### Standing long jump

This test entailed jumping forward as far as possible with feet separated at shoulder width and landing upright. A mattress with footprints was placed on the floor to direct participants to the jumping line. The participants were instructed to stand just behind the starting line. The distances between the starting line and the back heel of the participants were measured. Jumping with running was not counted. The participants took the test three times, resting in between each attempt. The best of three attempts was recorded in cm.

### Overarm beanbag throwing

The participants were instructed to throw a standard beanbag (Leisure and Cultural Department reference code: LC/TQ/55/14(P)) with 140 g using their preferred hand as far they could with an overarm posture. A 1000 cm/30 feet long place was required for the testing and every 150 cm/5 feet marked on the ground to facilitate the recording. The throwing distances (not including the sliding distance) of three attempts were measured. The recorded measurement for the farthest distance achieved in throwing the beanbag was in cm (greater or equal to 15 cm/6 in. counted as for one additional 12 in.).

### Anthropometric measurements

Participants' body weight and height were assessed using standard anthropometric techniques. Weight was measured with a portable digital scale (SECA 876, Hamburg, Germany) to the nearest 0.1 kg, while the participants were lightly dressed, wearing only a sport shirt. Height was measured to the nearest 0.1 cm while the children were standing upright in bare feet, using a portable stadiometer (SECA 217, Hamburg, Germany). The measurements were performed twice, and the average of the two measurements was recorded.

### Statistics

All statistical analyses were performed using R Statistical Software version 3.6.3 (http://cran.us.r-project.org/). The statistical tests were all conducted with a significance level of 0.05. Potential outliers were examined and removed if the fitness scores were ≥ 4 interquartile ranges from the group median by age and sex for each test.

Body mass index (BMI) was calculated as the weight in kilograms (kg) divided by the square of height in meters (m^2^). Students were categorized into underweight, healthy weight, and excessive weight (overweight/obese) groups based on their BMI according to the International Obesity Task Force (IOTF) age- and sex-specific standards^53^. The underweight group was defined using the international cut-off point, which aligns with the World Health Organization (WHO) standard of thinness, passing through a BMI of 17 at age 18. The excess weight (overweight/obese) group was categorized using the cut-off point passing through a BMI of 25 at age 18, while the remaining individuals were considered to be in the healthy weight group.

Descriptive statistics were employed to analyse the characteristics of the participants, which included age, sex, weight, height, BMI status, and motor test scores. To compare the mean differences of continuous variables such as age, weight, height, BMI, and motor test scores between boys and girls, an independent t-test was used. Pearson’s Chi-square test was used to examine the distribution of BMI status between the two sexes. One-way ANOVA was then used to compare the difference of the MP z-scores among different BMI status. Tukey HSD was followed to test for pairwise comparisons. MP z-scores were extracted from the generalized additive model for location, scale and shape GAMLSS models.

GAMLSS^54^, an extension of the LMS method developed by Cole and Green, was used to compute the normative values of the motor tests. The GAMLSS model consists of three components which are represented by four parameters: (1) location (µ), median; (2) scale (variability σ); (3) shape (skewness ν and kurtosis τ). Distributions available include normal distribution (NO) with only location and scale; Box-Cox-Cole-Green (BCCG) distribution with location, scale and skewness, where ν is equivalent to Box-Cox power λ; Box-Cox-power-exponential (BCPE) distribution which is an extension of BCCG distribution including kurtosis^18^.

The finesses of the models constructed using the four distributions mentioned above were compared using the Generalized Akaike Information Criterion (GAIC). The models were selected automatically using the LMS function of the GAMLSS package, which is implemented in R version 3.6.3. More information about the GAMLSS package can be found at http://gamlss.org/. The smallest GAIC with a penalty tree (GAIC(3)), which favours smoother curves, was chosen to provide an optimal fit for model^55^. Q-Q plot of the normalized quantile residuals^56^, Q statistics^57^ and worm plots^58^ were used to test the goodness of fit. Sex-specific sensitivity analysis was carried out by comparing the 5th, 10th, 20th, 30th, 40th, 50th, 60th, 70th, 80th, 90th and 95th percentile curves for the motor tests^18^.

### Informed consent

Informed consent was obtained from all subjects involved in the study.

## Data Availability

The datasets used and/or analysed during the current study are available from the corresponding author on reasonable request.

## Abbreviations

BMI: Body mass index
BT: Beanbag throwing
IOTF: International Obesity Task Force
WHO: World Health Organization
MP: Motor performance
OB: One-leg balance
SLJ: Standing long jump
